# Enhancing hazardous material vehicle detection with advanced feature enhancement modules using HMV-YOLO

**DOI:** 10.3389/fnbot.2024.1351939

**Published:** 2024-01-30

**Authors:** Ling Wang, Bushi Liu, Wei Shao, Zhe Li, Kailu Chang, Wenjie Zhu

**Affiliations:** ^1^Faculty of Computer and Software Engineering, Huaiyin Institute of Technology, Huaian, China; ^2^Nanjing University of Aeronautics and Astronautics Shenzhen Research Institute, Shenzhen, China

**Keywords:** hazardous material, vehicle detection, HMV-YOLO, spatial feature enhancement, LTPAN

## Abstract

The transportation of hazardous chemicals on roadways has raised significant safety concerns. Incidents involving these substances often lead to severe and devastating consequences. Consequently, there is a pressing need for real-time detection systems tailored for hazardous material vehicles. However, existing detection methods face challenges in accurately identifying smaller targets and achieving high precision. This paper introduces a novel solution, HMV-YOLO, an enhancement of the YOLOv7-tiny model designed to address these challenges. Within this model, two innovative modules, CBSG and G-ELAN, are introduced. The CBSG module's mathematical model incorporates components such as Convolution (Conv2d), Batch Normalization (BN), SiLU activation, and Global Response Normalization (GRN) to mitigate feature collapse issues and enhance neuron activity. The G-ELAN module, building upon CBSG, further advances feature fusion. Experimental results showcase the superior performance of the enhanced model compared to the original one across various evaluation metrics. This advancement shows great promise for practical applications, particularly in the context of real-time monitoring systems for hazardous material vehicles.

## 1 Introduction

Hazardous material vehicle detection plays a crucial role in ensuring public safety and minimizing the risks associated with the transportation of dangerous goods. Accurate and efficient detection systems are essential to prevent accidents, respond to emergencies, and safeguard the environment (Landucci et al., 2017). Various computer vision techniques and deep learning models have been employed to enhance the accuracy of hazardous material vehicle detection, significantly improving the ability to identify and mitigate potential threats posed by such vehicles (Wang et al., 2018).

In recent years, vehicle detection methods based on deep learning have been gradually gaining prominence (Maity et al., 2021). These methods possess the ability to automatically learn features from data, leading to outstanding detection performance in various scenarios. Deep learning-based vehicle detection methods can be categorized into two main types: two-stage detection methods and one-stage detection methods. Among the two-stage detection methods, the R-CNN series of models (Girshick et al., 2014; Girshick, 2015; Ren et al., 2015) stand out as particularly excellent representatives. One-stage object detection methods like YOLO (Redmon et al., 2016) have been widely used for dense predictions at every position in the feature map without the need for additional region proposal steps. Bochkovskiy et al. (2020) introduced several new methods to improve the accuracy of CNN, such as WRC, CSP, CmBN, SAT, and Mish activation functions, and combined them to achieve 43.5% AP and 65 FPS on the MS COCO dataset. Li et al. (2022) extensively studied the latest advances in object detection, including network design, training strategies, test techniques, quantification, and optimization methods, and integrated them into the built YOLOv6. YOLOv6-N achieved 35.9% AP on the COCO dataset, outperforming other mainstream target detectors of the same size. Wang et al. (2023) proposed a trainable bag-of-freebies solution. Combining a flexible and efficient training tool with the proposed architecture and composite scaling method, YOLOv7 outperforms all known target detectors in both speed and accuracy. These methods often employ various techniques to address different requirements in various detection scenarios, making one-stage methods simpler and more efficient. On the other hand, two-stage methods involve explicit region proposal and classification/localization stages, which can achieve higher accuracy in some cases but may be slightly slower in terms of speed. One-stage methods achieve faster detection by directly predicting object attributes. Therefore, one-stage methods are more widely applied compared to two-stage methods.

Given the prevailing trends favoring the efficiency of one-stage methods, especially in real-time applications, it becomes evident that their direct prediction of object attributes aligns seamlessly with the need for prompt detection. However, in hazmat vehicle detection, where accuracy is paramount, a nuanced approach is essential. Our research strategically navigates this landscape by adapting the one-stage paradigm, as exemplified by the YOLO architecture, to strike a balance between speed and precision specifically tailored for hazmat scenarios. The contributions of this paper are as follows:

- Two innovative modules are proposed and systematically evaluated: Convolutional Block with SiLU and Global Response Normalization (CBSG) and G-ELAN. These modules incorporate global response normalization into traditional convolutional layers with the aim of mitigating feature collapse and boosting neuron activity.
- A novel structure called Long-Range Temporal Pyramid Attention Network (LTPAN) is introduced to enhance feature interaction between deep and shallow networks, thereby improving the model's feature fusion capability.
- The paper provides a detailed description of the stepwise integration of these modules into the YOLOv7-tiny model and presents a comprehensive evaluation of their impact on various performance metrics. This analysis aims to contribute to improved safety and security in the transportation of hazardous materials.

## 2 Related work

### 2.1 Traditional object detection method

Traditional methods in the early stages heavily relied on manually designed feature extractors, such as Histogram of Oriented Gradients (HOG) (Dalal and Triggs, 2005). These features were capable of capturing information about the shape, edges, and other relevant characteristics of vehicles. Li and Guo (2013) introduced a single-camera front vehicle detection method based on HOG features and SVM, incorporating vehicle shadow features, which demonstrated accurate vehicle identification under varying daylight conditions. Han et al. (2006) proposed a two-stage approach that used stereo vision cues to generate potential object positions and then employed extended HOG features and SVM classifiers to verify all hypotheses, enabling the identification of both people and vehicles with high detection accuracy while achieving faster processing speeds. Cao et al. (2011) presented a mobile vehicle detection method based on enhanced HOG features, overcoming challenges posed by lighting variations and scene complexity. Compared to traditional methods, this approach exhibited superior performance in terms of detection rate, false positive rate, and processing speed. Cheon et al. (2012) introduced a visual detection method that completed vehicle detection through hypothesis generation and hypothesis verification steps. This method demonstrated robust performance in experiments, contributing significantly to vehicle detection, particularly in scenarios where deep learning methods may not be readily applicable due to data constraints or resource limitations.

In addition to traditional computer vision-based methods, there are also sensor-based approaches in vehicle detection. Petrovskaya and Thrun (2009) proposed a mobile vehicle detection and tracking module based on a laser rangefinder platform that used a single Bayesian filter to model and estimate the dynamic and geometric properties of vehicles. It also introduced the concept of motion evidence to overcome low signal-to-noise ratio challenges when detecting moving vehicles in urban environments. This approach enabled the efficient creation of 2D representations and detection of hard-to-identify black vehicles. Yang and Lei (2014) proposed a vehicle detection and classification system based on magnetoresistive sensors, utilizing a novel fixed threshold state machine algorithm to detect vehicles and classify them based on the time they spent entering and exiting the sensor monitoring area. It addressed the problem of detecting and classifying vehicles in slow-moving traffic conditions. Leitloff et al. (2010) presented an automatic vehicle detection method from optical satellite images that used adaptive feature enhancement to generate single-vehicle target hypotheses, combined with line extraction to detect vehicle queues, and achieved vehicle detection tasks through robust parameter estimation. Ali et al. (2011) introduced a novel inductive loop sensor suitable for heterogeneous and lane-less guided traffic scenarios, allowing for the classification and accurate counting of common vehicles. This approach adapts to various traffic situations and provides alternative approaches to vehicle detection, each with its advantages and applicability in specific scenarios.

### 2.2 Object detection algorithm based on deep learning

In recent years, detection methods based on deep learning have gradually emerged, demonstrating excellent detection performance in various scenarios by automatically learning features from data. Nagarajan and Gopinath (2023) proposed an indoor object detection method called HAAVO, which utilizes generative adversarial networks and deep convolutional neural networks for target detection and recognition. Simultaneously, it optimizes the training of DCNN classifiers and deep residual networks to estimate distance. Additionally, HAAVO integrates the Honey Badger Algorithm, Adam Optimizer, and African Vultures Optimization. The method exhibits outstanding performance in terms of test accuracy, precision, and recall. Dewangan and Sahu (2023) introduced a two-tier lane detection framework based on deep learning. This framework extracts texture features based on local binary patterns and employs an optimized deep convolutional neural network for classification. Weight fine-tuning is performed using the Flight Straight of Moth Search (FS-MS) Algorithm. Compared to other improved CNN methods of the same type, this framework effectively enhances computational efficiency. Furthermore, Dewangan and Sahu (2022) proposed a road detection model based on Siamese Fully Convolutional Network (s-FCN-loc). This model combines semantic contours, RGB channels, and prior location information, achieving precise segmentation of road areas. The Distance-guided Sea Lion Optimization (DSLnO) algorithm is employed to optimize the selection of convolutional layers in the FCN network, thereby improving detection accuracy. The method demonstrates accuracy and performance superior to traditional approaches on the KITTI road detection dataset. Chen et al. (2022b) proposed a garbage classification method called GCNet, which enhances the ShuffleNet v2 architecture by introducing parallel mixed attention mechanisms, new activation functions, and transfer learning techniques. Experimental results demonstrate that GCNet achieves an average accuracy of 97.9% on a self-built dataset, providing effective support for machine vision technology in the fields of garbage classification and resource recycling. Additionally, Chen et al. (2023) introduced a railway track region segmentation network (ERTNet) based on an encoder-decoder architecture. This method employs deep convolution and channel shuffling to construct lightweight feature extraction units, combines a feature-matching cross-fusion decoder with knowledge distillation techniques to enhance segmentation accuracy. Simultaneously, a loss function is proposed to penalize pixels with large offsets, achieving efficient track region segmentation. Experimental results indicate that this approach achieves high segmentation accuracy while ensuring efficient computational performance.

In two-stage object detection methods, Luo et al. (2021) proposed a vehicle detection model based on Faster R-CNN, which effectively detects vehicles of multiple scales in traffic scenes through NAS optimization. This model initially enhances image quality using the RIAC algorithm, then utilizes NAS to generate optimal cross-layer connections for efficient feature extraction across multiple layers. Finally, it employs object feature enrichment methods in combination with contextual information to enhance the information about vehicle targets. This approach improves the robustness of detection, particularly for small-scale and occluded targets. Beery et al. (2020) proposed Context R-CNN, a method based on attention mechanisms that leverage temporal context features from unannotated frames in the camera to improve target detection performance. Additionally, this method can index a long-term memory bank and aggregate context features from other frames to enhance target detection performance in the current frame. Nguyen (2019) introduced an improved vehicle detection method based on Faster R-CNN, which incorporates technologies such as MobileNet, Soft NMS, context-aware RoI pooling layers, and depthwise separable convolution to enhance the accuracy and efficiency of vehicle detection. Li et al. (2019) presented the Stereo R-CNN model for 3D object detection, an extension of Faster R-CNN that fully exploits feature information from stereo images. While performing detection, this model can also associate left and right target information in stereo images, effectively improving detection accuracy. These deep learning-based methods have made significant strides in vehicle detection, offering improved accuracy and robustness in various application scenarios.

Single-stage detection methods, when compared to two-stage detection methods, offer faster detection speeds and more accurate bounding boxes. These methods directly predict the location and category of objects from input images without the need for region proposal selection and classification, thereby effectively reducing inference time. Additionally, single-stage detection methods, exemplified by the YOLO series (Redmon et al., 2016), enhance their ability to detect objects of varying sizes and shapes through end-to-end training and multi-scale feature utilization, resulting in improved detection performance. Chen et al. (2022a) proposed an enhanced SSD algorithm for vehicle detection. The algorithm employs MobileNet v2 as the feature extraction network and achieves feature weighting and bottom-up feature fusion through channel attention mechanisms and deconvolution modules, thereby enhancing detection accuracy. Experimental results demonstrate that the algorithm improves both inference speed and prediction accuracy, providing an effective solution to the vehicle detection problem in autonomous driving systems. Feng et al. (2023) introduced an improved YOLOv5s algorithm, replacing the neck of YOLOv5s with a slim-neck and utilizing Ghost-Shuffle Conv and VoV-GSCSP. This modification aims to reduce computational and network complexity while maintaining sufficient accuracy. Furthermore, knowledge distillation is applied to optimize the enhanced YOLOv5s model, enhancing its generalization ability and overall performance. Experimental results show that the algorithm provides real-time, high-precision detection of small winter jujube fruits for robotic applications. Dong et al. (2022) presented an improved lightweight YOLOv5 method for vehicle detection, incorporating C3Ghost and Ghost modules to reduce computational complexity and enhance feature representation. Additionally, it uses CBAM for selecting essential information and suppressing unimportant details. Moreover, it adopts CIoU Loss as the bounding box regression loss function. This approach enhances detection accuracy while reducing computational demands and model parameters compared to existing methods. Zhang et al. (2023) enhanced the YOLOv7 backbone network with a Res3Unit structure to improve the model's ability to capture nonlinear features. They introduced the ACmix mixed attention mechanism to increase the network's focus on vehicles and reduce interference from other objects. Finally, Gaussian receptive fields are employed to enhance the model's sensitivity to small objects in images. These enhancements collectively lead to improved accuracy and speed in vehicle detection on urban roads. Qiu et al. (2023) introduced an algorithm called YOLO-GNS for detecting special vehicles from a drone's perspective. This algorithm enhances feature extraction capabilities by introducing the Single Stage Headless (SSH) structure, making it suitable for detecting small or ambiguous objects. Additionally, it draws inspiration from the GhostNet concept, simplifying complex convolutions into linear transformations to reduce computational costs, thereby improving the average detection accuracy. Lin and Jhang (2022) proposed an intelligent traffic monitoring system based on YOLO and convolutional fuzzy neural networks (CFNN). Initially, the system employs the YOLO algorithm to detect vehicles. It then combines vehicle counting methods to calculate traffic flow. Subsequently, two models, CFNN and Vector-CFNN, combined with a network mapping fusion method, are introduced to improve detection accuracy and real-time performance.

In conclusion, the single-stage detection methods discussed in the preceding text offer faster detection speeds and more precise bounding box predictions compared to their two-stage counterparts. Leveraging end-to-end training, multi-scale feature utilization, and innovative enhancements from the YOLO series result in superior detection performance.

### 2.3 Comparison of related methods

In general, methods for vehicle detection can be categorized into feature extractor methods, sensor-based methods, traditional deep learning methods, two-stage object detection methods, and one-stage object detection methods. This paper conducts a comparative analysis of these methods, as summarized in Table 1.

**Table 1:** Comparison of vehicle detection methods.

**Method categories**	**Common methods**	**Major advantage**	**Major limitations**
Feature extractor	HOG, etc.	Strong generality	Sensitive
Sensors	Radar, etc.	Adaptable	High cost
Traditional CNNs	ResNet, etc.	Scalable	Resource-intensive
Two-stage detectors	RCNN, etc.	Accurate	Complex
One-stage detectors	YOLO, etc.	Real-time	Limited precision

In methods based on feature extractors, representations like HOG are commonly encountered. These methods achieve target detection by extracting local features from images, such as gradient directions. While they exhibit excellent performance in handling simple scenes, their effectiveness may be limited in complex and dynamically changing environments. In contrast, sensor-based methods integrate various types of sensors, such as cameras, millimeter-wave radar, and lidar, to acquire multimodal information and enhance the system's perception of the environment. However, this integration brings about challenges, including higher costs and difficulties in data fusion. Additionally, there is a growing trend in the adoption of traditional deep learning methods, with common models such as ResNet and EfficientNet capable of automatically learning complex features. Nevertheless, deep learning methods demand a substantial amount of annotated data, and their black-box nature may complicate the interpretation of the model's decision-making process.

In two-stage object detection methods, the R-CNN series models are renowned for their high accuracy but are relatively slower in speed. On the other hand, single-stage object detection methods, exemplified by SSD and the YOLO series, are more suitable for real-time scenarios, even though their accuracy may be relatively lower when dealing with small targets and complex scenes.

While numerous deep learning-based vehicle detection models exist, those specifically tailored for hazardous material (hazmat) vehicles are relatively scarce, and existing approaches often suffer from subpar average precision. To address these challenges, we introduce a novel hazmat vehicle detection model based on YOLOv7-tiny. This model offers improved accuracy for detecting hazmat vehicles and is well-suited for real-world applications.

## 3 Proposed method

**Figure 1:**
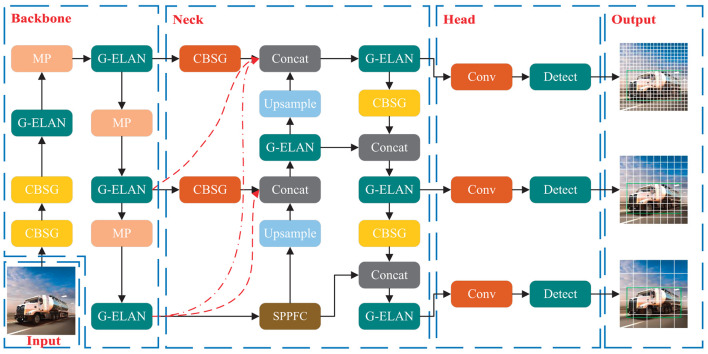
Architecture of the HMV-YOLO model.

Figure 1 delineates the architecture of the HMV-YOLO model meticulously tailored for hazardous material vehicle detection. The incorporation of two innovative modules, CBSG and G-ELAN, stands as a testament to the model's advancements. These modules play a pivotal role in seamlessly integrating global response normalization into conventional convolutional modules, strategically addressing the pervasive issue of feature collapse. Simultaneously, they actively amplify neuron activity within the network, enhancing the effectiveness of extracting features related to dangerous chemical vehicles and improving the overall performance of the model.

Furthermore, the model introduces a groundbreaking structural component known as LTPAN, strategically designed to elevate the interaction of features between deeper and shallower layers of the network. This deliberate enhancement significantly amplifies the model's capability to fuse and synergize features crucial for the precise detection of hazardous material vehicles. The intricate combination of modules and structural advancements positions the HMV-YOLO model as a superior solution in the realm of detecting dangerous chemicals within vehicles.

### 3.1 CBSG and G-ELAN

To address redundancy in feature maps, the approach did not directly remove them from the network. Instead, they were utilized to learn more effective features. The ConvNext V2 approach (Woo et al., 2023) was adopted for this purpose, introducing the concept of Global Response Normalization (GRN). The primary objective of GRN is to elevate channel contrast and selectivity, achieved through three distinct steps: global feature aggregation, feature normalization, and feature calibration.

A set of aggregated values $G\left(X\right)=gx=\left\{\left||X_{1}||,||X_{2}||,...||X_{C}|\right|\right\}\in R^{C}$ is obtained through the global function. For the input feature *X*∈*R*^*H*×*W*×*C*^ GRN first aggregates the spatial feature maps *X*_*i*_ into the vector using the global function *G*(·) as Equation 1:


(1) $$G(X):X\in R^{H\times W\times C}\to gx\in R^{C}$$

Where *G*(*X*)_*i*_ = ||*X*_*i*_|| represents scalar statistics information aggregated for the *i*-th channel.

Next, the aggregated values are processed using the response normalization function *N*(·), as Equation 2:


(2) $$N(∥X_{i}∥):∥X_{i}∥\in R\to \frac{∥X_{i}∥}{\sum_{j=1,...C}∥X_{j}∥}\in R$$

Where ||*X*_*i*_|| is the L2 norm of the *i*-th channel. Finally, the computed feature normalization scores are used to calibrate the original input response, as Equation 3:


(3) $$X_{i}=X_{i}*N(G{\left(X\right)}_{i})\in R^{H\times W}a$$

Furthermore, two learnable parameters, α and β initialized to zero, are introduced in the Global Response Normalization to simplify optimization. The final equation can be expressed as Equation 4:


(4) $$X_{i}=\gamma *X_{i}*N(G{\left(X\right)}_{i})+\beta +X_{i}$$

In the model, GRN is applied after the activation function within the CBS module, giving rise to a new convolutional module named CBSG. Simultaneously, the YOLOv7-tiny architecture undergoes the replacement of the CBS module with the CBSG module, forming what is now referred to as the new G-ELAN.

### 3.2 LTPAN

In the network's neck, YOLOv7-tiny utilizes a Path Aggregation Network (PAN) (Liu et al., 2018) to facilitate information exchange and feature fusion between feature maps at different levels. However, PAN's fusion approach, involving only bottom-up and top-down paths, proves simplistic and falls short in capturing features of hazardous material vehicles across varying-sized feature maps. This limitation results in an insufficient representation of features with rich semantic and multi-scale information.

**Figure 2:**
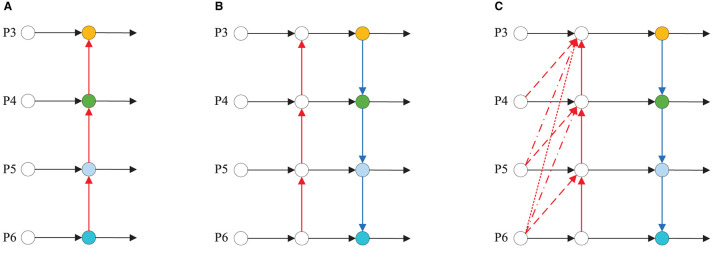
Different neck structures. **(A)** FPN structure. **(B)** PAN structure. **(C)** LTPAN structure.

To address this issue, we propose a novel LTPAN structure, illustrated in Figure 2. In the figure, (a) shows the FPN structure, (b) shows the PAN structure, (c) shows the LTPAN structure, red dashed lines with varying styles represent different magnitudes of upsampling operations. To enhance the model's ability to represent diverse features, we introduce a 1 × 1 convolutional layer before each upsampling operation to linearly combine features from different channels. Considering that hazardous material vehicles in images are typically larger objects, and feature maps of different sizes in the backbone network often contain richer semantic information, the LTPAN structure incorporates a novel low-dimensional feature mapping structure. This structure maps feature maps of different sizes from the backbone network to the feature maps at the network's neck through upsampling.

### 3.3 Loss function

The loss function of HMV-YOLO consists of confidence loss *l*_*obj*_, classification loss *l*_*cls*_, and bounding box position loss *l*_*box*_. The network partitions the feature map into multiple cells, with each cell corresponding to a vector *y* = (*t*_*x*_, *t*_*y*_, *t*_*w*_, *t*_*h*_, *p*_*o*_, *c*_1_, *c*_2_, *c*_3_, *c*_4_), where *t*_*x*_ and *t*_*y*_ represent the offsets between the predicted box and its corresponding anchor box, while *t*_*w*_ and *t*_*h*_ represent the width and height of the predicted box. *p*_*o*_ represents the probability that the cell contains an object, and *c*_1_, *c*_2_, *c*_3_, *c*_4_ are the predicted values for the four categories in the dataset. The loss function is calculated as Equation 5:


(5) $$\begin{array}{l} L(t_{p},t_{gt})=\sum_{k=0}^{K}[\alpha_{k}^{balance}\alpha_{box}\sum_{i=0}^{S^{2}}\sum_{j=0}^{B}\prod_{kij}^{obj}L_{CIoU}+\\ \alpha_{obj}\sum_{i=0}^{S^{2}}\sum_{j=0}^{B}\prod_{kij}^{obj}L_{obj}+\alpha_{cls}\sum_{i=0}^{S^{2}}\sum_{j=0}^{B}\prod_{kij}^{obj}L_{cls}] \end{array}$$

The confidence loss *l*_*obj*_ is determined based on positive sample matching, which includes the object confidence score *p*_*o*_within the predicted box and the intersection over union between the predicted box and the Ground Truth. Both of these terms are computed using binary cross entropy to obtain the final object confidence loss. The confidence loss *l*_*obj*_ is defined as Equation 6:


(6) $$\begin{array}{l} l_{obj}=\sum_{i=0}^{S^{2}}\sum_{j=0}^{B}l_{ij}^{obj}\left(\hat{C_{i}}log(C_{i})+(1-\hat{C_{i}})log(1-C_{i})\right)-\\ \;\lambda_{nobj}\sum_{i=0}^{S^{2}}\sum_{j=0}^{B}l_{ij}^{nobj}\left({\hat{C}}_{i}log(C_{i})+(1-{\hat{C}}_{i})log(1-C_{i})\right) \end{array}$$

The classification loss *l*_*cls*_ is similar to the confidence loss *l*_*obj*_ and is calculated based on the class score of the predicted box and the one-hot encoded class of the Ground Truth box. It is defined as Equation 7:


(7) $$l_{cls}=\sum_{i=0}^{S^{2}}l_{ij}^{obj}\sum_{c\in classes}({\hat{P}}_{i}(c)log(P_{i}(c))+(1-{\hat{P}}_{i}(c))log(1-P_{i}(c)))$$

The bounding box position loss is used to measure the difference between the predicted box and the true box, taking into account overlap area, centroid distance, and aspect ratio. It is calculated using the CIoU Loss, as Equations 8 and 9:


(8) $$l_{b\alpha x}=l_{CloU}=1-CIoU=1-(IoU-\frac{d_{o}^{2}}{d_{c}^{2}}-\frac{\nu^{2}}{1-IoU+\nu })$$

(9) $$\nu =\frac{4}{\pi^{2}}{\left(arctan\frac{w^{gt}}{h^{gt}}-arctan\frac{w}{h}\right)}^{2}$$

Where *d*_*o*_ is the Euclidean distance between the bounding box and the Ground Truth box centroids, *d*_*c*_ is the diagonal distance between the bounding boxes, *v* is a parameter that measures aspect ratio consistency, *w*^*gt*^ and *h*^*gt*^ are the width and height of the Ground Truth box, and *w* and *h* are the width and height of the predicted box.

## 4 Result and discussion

### 4.1 Simulation environment and parameters

In this experiment, we utilized a custom dataset, annotating collected images with the Labelimg tool. The targets were categorized into four types: car, bus, truck, and hazardous material vehicle, based on vehicle types in the images. Ultimately, we obtained a total of 3,490 annotated images, with those containing hazardous material vehicles representing over 70% of the dataset.

To ensure the reliability of our experimental data, all experiments were conducted under consistent hardware settings and parameter configurations. Model training, parameter optimization, and updates were performed using two Tesla V100 16GB GPUs with the stochastic gradient descent (SGD) algorithm. The input image size was set to 640 × 640 × 3, batch size to 64, initial learning rate to 0.01, and the total number of training epochs to 200.

### 4.2 Model validation

To determine the most effective pairing of GRN and activation function, combination experiments were conducted using various activation functions. The outcomes are presented in Table 2, demonstrating that optimal results are achieved when GRN is combined with the SiLU activation function.

**Table 2:** Experimental results of combinations of GRN with different activation functions.

	**ReLU**	**SiLU**	**LeakyReLU**	**SELU**	**GELU**	**ELU**	**Tanh**
map@.5	0.846	0.856	0.838	0.833	0.841	0.849	0.8

Bold values represents the best metrics.

**Figure 3:**
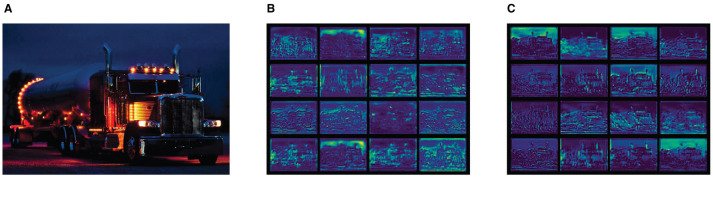
Output feature maps of different modules. **(A)** Input image. **(B)** Output feature map from CBS. **(C)** Output feature map from CBSG.

During the visual analysis of the trained YOLOv7-tiny model (the input image is Figure 3A), instances of feature collapse within specific feature maps were identified (Figure 3B). These feature maps were characterized by redundant and uninformative feature mappings, contributing minimally to the model's performance and resulting in an accumulation of surplus redundant information.

The visualization of the feature maps associated with the enhanced CBSG module at the same location is shown in Figure 3C. In contrast to the original CBS module, the CBSG module effectively mitigated feature collapse, presenting improved feature maps. This enhancement facilitated the network in capturing more valuable and informative features, contributing to an overall improvement in performance.

The CBSG module offers notable advantages across multiple dimensions. By alleviating feature collapse, it refines feature information, making the content in the feature maps more representative. Additionally, it enhances performance by diminishing the impact of redundant information, improving the accuracy and efficiency of target detection. The introduction of the CBSG module also bolsters the model's robustness during training, rendering it more adaptable to various complex scenarios.

**Figure 4:**
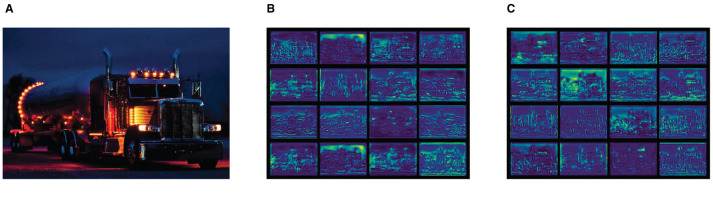
Output feature maps of different structures. **(A)** Input image. **(B)** Output feature map of original model. **(C)** Output feature map after incorporating the LTPAN structure.

To investigate the impact of LTPAN on the network's feature extraction capability, we generated feature maps preceding the prediction head by integrating the LTPAN structure, as illustrated in Figure 4 [(a) is the input image, (b) is the output feature map of original model, (c) is the Output feature map after incorporating the LTPAN structure]. Evidently, the feature maps associated with the LTPAN structure exhibit a significantly broader range of feature information compared to the original model. This observation confirms the effectiveness of incorporating the LTPAN architectural addition.

The enhanced feature extraction provided by LTPAN is instrumental in capturing nuanced details and diverse contextual information. The augmented feature maps contribute to a more comprehensive representation of the input data, empowering the network with richer and more discriminative features. This enhancement is particularly valuable in scenarios where objects exhibit intricate patterns or are situated in complex backgrounds.

Furthermore, the broader feature coverage achieved by LTPAN is advantageous for handling scale variations and addressing instances where objects may appear at different sizes within the input images. This adaptability is crucial for robust object detection across diverse datasets and real-world scenarios.

To confirm the efficacy of the model enhancements, a comparative analysis was conducted against prevalent two-stage and one-stage models currently in use. The detailed results of these experiments are presented in Table 3 for thorough examination.

**Table 3:** Experimental results from different models.

**Methods**	***P***	***R***	**map@.5**	**map@.5.95**
Faster R-CNN	0.834	0.769	0.717	0.451
YOLOv3	0.726	0.689	0.705	0.416
YOLOv4	0.857	0.756	0.837	0.654
YOLOv5s	0.919	0.761	0.847	0.639
YOLOv6n	0.919	0.761	0.853	0.69
YOLOv7-tiny	0.816	0.789	0.833	0.639
YOLOv8n	**0.941**	0.752	0.843	**0.696**
HMV-YOLO	0.865	**0.801**	**0.863**	0.663

Bold values represents the best metrics.

From the table, it is apparent that the model outperforms its counterparts across most metrics, with a notably substantial improvement in the *map@*.5 metric. It surpasses the top-performing model by 1.1% and even outperforms the latest YOLOv8 by 2%. Although the *Precision* metric does not achieve the best result, the model's *Recall* stands as the highest among all models, signifying that the approach detects a greater number of objects, highlighting its effectiveness.

In comparison to the original model, this approach manifests comprehensive improvements across various metrics. Notably, there's a 4.9% increase in *Precision*, a 1.2% increase in *Recall*, a 3.0% improvement in *map@*.5, and a 2.4% improvement in *map@*.5.95. These improvements across diverse metrics underscore how the amalgamation of enhanced modules can significantly boost the model's efficiency.

The CBSG module plays a pivotal role in suppressing feature collapse, allowing the network to acquire more informative feature details. Simultaneously, the LTPAN structure facilitates the fusion of richer features from the deep and shallow networks, with a particular emphasis on features associated with larger objects.

**Figure 5:**
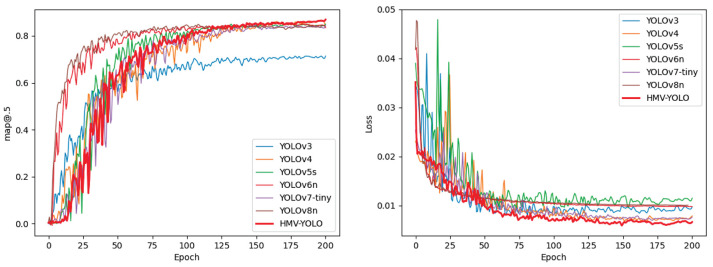
Map curve **(left)** and loss curve **(right)**.

Map curve analysis provides insights into the performance evolution of object detection models during the training process. In Figure 5 (left), the changing trends of mAP for each model are presented as the number of training epochs increases. The *x*-axis represents the growth of training epochs, the *y*-axis represents the growth of mAP, and the curves depict the changing trends of mAP for each model during training. By observing the curves, we can distinguish the evolving trends of mAP for each model during the training process.

YOLOv6n and YOLOv8n exhibit higher mAP values in the early stages of training, suggesting a quicker adaptation and learning ability to the task. YOLOv3 reaches a bottleneck in mAP growth around 70 epochs, showing a stable trend afterward, while other models gradually reach their peaks in subsequent epochs. The mAP curve of HMV-YOLO demonstrates a unique trend, surpassing all other models around 130 epochs, showcasing a stronger continuous learning ability and reflecting its special advantages in hazardous material vehicle detection tasks.

The loss curves in Figure 5 (right) provide insights into the learning processes of different object detection models. By observing the curves, YOLOv4 and YOLOv5s exhibit significant loss fluctuations in the early stages of training, while YOLOv6n and YOLOv8n show a relatively stable learning process. YOLOv3 and YOLOv7-tiny demonstrate a comparatively slower learning speed in the initial phases, whereas the loss curve of HMV-YOLO follows a unique trend, achieving the lowest loss among all models in a stable manner after ~50 training epochs.

In many cases, the enhancement of various model metrics often accompanies a significant increase in parameters. However, trading a substantial parameter increase for slight metric improvement may not be a prudent choice. To assess this trade-off, we compared the *Parameters*, Floating Point Operations (*FLOPs*), and *Volume* of several mainstream models with our proposed model, as detailed in Table 4.

**Table 4:** Comparison of model characteristics: parameters, FLOPs, volume, and Inf time.

**Methods**	**Image size**	**Parameters**	**Flops**	**Volume**	**Inf time**
YOLOv3-tiny	640 × 640	12.1 M	19.0G	23.23 MB	22.3 ms
YOLOv4-tiny	640 × 640	6.0 M	16.5G	46.30 MB	35.7 ms
YOLOv5s	640 × 640	7.0 M	16.0G	13.81 MB	20.2 ms
YOLOv6n	640 × 640	4.7 M	11.4G	9.96 MB	17.3 ms
YOLOv7-tiny	640 × 640	6.0 M	13.2G	11.75 MB	20.8 ms
YOLOv8s	640 × 640	11.1 M	28.6G	85.44 MB	29.2 ms
HMV-YOLO	640 × 640	6.3 M	13.6G	12.04 MB	19.6 ms

The results indicate that our proposed model, in comparison to YOLOv7-tiny, experiences only a 0.4G increase in *FLOPs*, a mere 0.3 M increase in *Parameters*, and a minimal 0.28MB increase in *Volume*. While our model may not achieve the fastest processing speed in terms of inference time, it still demonstrates a 1.2 ms improvement compared to YOLOv7-tiny. Considering information from other models, our proposed model in this paper remains highly competitive.

To vividly illustrate the enhanced performance of the model, a comprehensive breakdown of *Precision*, *Recall*, *map@*.5, and *map@*.5.95 for each category within the dataset is provided. This comparison against the original model is thoughtfully presented in Table 5 for thorough examination.

**Table 5:** Experimental results for various categories in the dataset.

	**YOLOv7-tiny**				**HMV-YOLO**			

**Classes**	*P*	*R*	**map@.5**	**map@.5.95**	*P*	*R*	**map@.5**	**map@.5.95**
HMV	0.936	0.918	0.96	0.824	**0.944**	**0.919**	**0.967**	**0.843**
Truck	0.733	**0.717**	0.734	0.522	**0.829**	0.685	**0.757**	**0.574**
Bus	0.886	0.77	0.853	0.664	**0.902**	**0.838**	**0.912**	**0.719**
Car	0.709	0.751	0.787	**0.546**	**0.785**	**0.762**	**0.817**	0.542

Bold values represents the best metrics.

**Figure 6:**
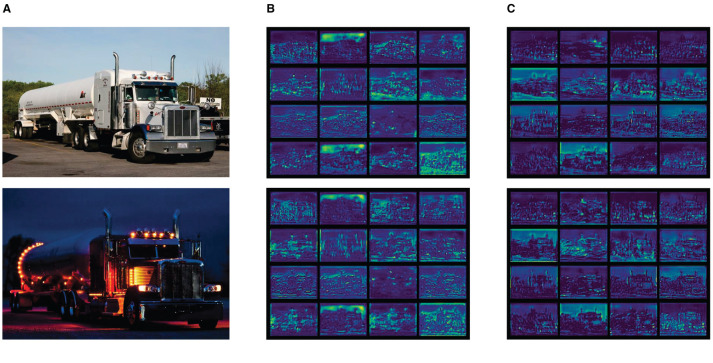
Output feature maps of different models. **(A)** Input image. **(B)** Output feature map of YOLOv7-tiny. **(C)** Output feature map of HMV-YOLO.

Moreover, a meticulous examination is conducted on a curated set of images encompassing both daytime and nighttime scenarios. This analysis involves extracting feature maps with dimensions of 80 × 80 from both the original and improved models. The comparative evaluation of these feature maps is visually depicted in Figure 6, enabling a direct side-by-side assessment.

The visual representation unmistakably highlights the considerable superiority of the enhanced approach over the original model. The feature maps derived from the improved model exhibit enhanced clarity, richer details, and a more pronounced ability to capture relevant features across diverse lighting conditions. This superiority is particularly evident in nighttime scenarios, where the enhanced model demonstrates superior adaptability and feature representation.

These experimental findings reinforce the efficacy of the proposed enhancements, showcasing consistent performance improvements across varying environmental conditions. The visually compelling evidence from Figure 6 underscores the robustness and versatility of the improved model, positioning it as a formidable solution for object detection tasks in both daytime and nighttime scenarios.

An examination of various initial learning rates on the enhanced model's performance was conducted. The results, as presented in Table 6, reveal an interesting trend: an increase in the initial learning rate correlates with slight improvements across most metrics. Notably, within the range of initial learning rates between 0.02 and 0.04, the model consistently achieves superior results across all metrics, demonstrating the model's responsiveness to this specific parameter adjustment.

**Table 6:** Impact of different initial learning rates.

**Init LR**	***P***	***R***	**map@.5**	**map@.5.95**
0.01	0.865	0.801	0.863	0.663
0.02	**0.923**	0.766	0.865	0.678
0.03	0.871	**0.809**	**0.876**	0.686
0.04	0.9	0.785	0.873	**0.687**
0.05	0.882	0.79	0.86	0.678
0.06	0.882	0.79	0.858	0.676

Bold values represents the best metrics.

### 4.3 Detection performance analysis

To explore the model's ability to generalize, a set of images with various dimensions from external sources was gathered. Detection experiments were conducted in two distinct scenarios: multi-object and multi-class situations. The primary objective was to evaluate the detection performance of the enhanced model under different conditions. The experiment employed distinct bounding box colors for visual representation to facilitate class differentiation, with hazardous material vehicles indicated by orange bounding boxes, trucks by pink, cars by yellow, and buses by red.

**Figure 7:**
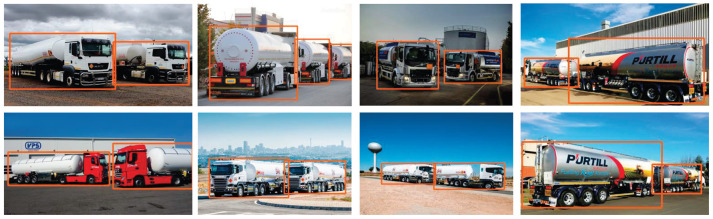
Detection results in multi-object scenarios.

In the multi-object scenario, the LTPAN structure played a crucial role in improving the model's performance by optimizing the fusion of pertinent feature information for larger targets, particularly hazardous material vehicles. This optimization involved categorizing different target features into distinct feature layers for more effective processing. The results, depicted in Figure 7, exemplify the model's exceptional accuracy, even successfully identifying a vehicle towing two separate hazardous material tanks.

**Figure 8:**
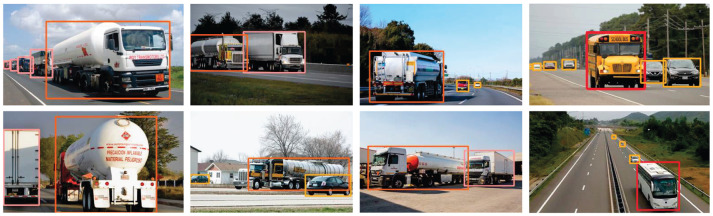
Detection results in multi-class scenarios.

Real-world applications frequently present diverse challenges, including targets belonging to multiple classes, variations in size, and varying degrees of occlusion. These challenges rigorously test the model's ability to generalize. To address this, Global Response Normalization was introduced to enhance neuron activation. This enhancement empowers the model to attentively process all regions of the feature maps, detect smaller and partially occluded targets, and extract more precise class information. Consequently, this enriches the model's ability to generalize, as evident in the detection results showcased in Figure 8.

**Figure 9:**
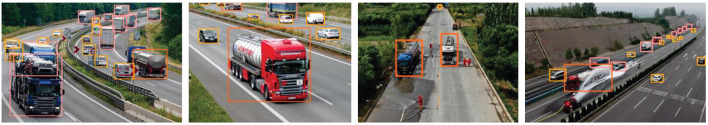
Detection results in monitoring perspective.

In the experiment analyzing detection performance, this paper extends the evaluation of the object detection model's performance, examining scenarios from the perspectives of road surveillance and drone aerial views, as shown in Figure 9. The first image displays a congested highway where the model performs remarkably well, with clear and accurate boundaries of detected objects. The model can precisely identify and locate moving vehicles, even when a large truck in the lower-left corner is partially obscured by vehicles in the front.

The second image illustrates a typical road scene, presenting a challenge with a hazardous material vehicle in the upper-left corner where feature loss occurs due to camera field of view issues. Despite this challenge, the model accurately detects the hazardous material vehicle.

The third image presents an aerial view from a drone, requiring the model to handle the deformation of vehicles and changes in other objects on the road. The model continues to perform well in this scenario, successfully detecting various objects and providing accurate localization.

The last image demonstrates the detection results of low-resolution drone aerial images, where some objects may lack clarity due to image quality loss and obstacles. The model maintains a certain level of detection accuracy under these conditions, but additional optimization may be needed, especially in the context of low-resolution aerial views, to ensure precise capture of small targets.

**Figure 10:**
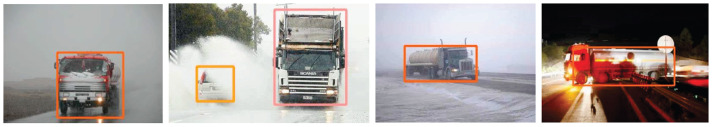
Detection results in different environmental.

In addition to considering different shooting angles, we further explored the model's performance under various environmental conditions to comprehensively assess its robustness and adaptability in different meteorological environments. As shown in Figure 10, the first and second images depict rainy weather conditions, demonstrating the model's strong robustness in the presence of raindrop obstructions and wet road surfaces. Even in conditions with blurred visibility, the model accurately detects hazardous material vehicles on the road and can even detect vehicles obscured by water splashes, showcasing its adaptability in wet weather conditions.

The third image represents snowy weather conditions accompanied by some fog. The model successfully handles the image noise caused by snowflakes and fog, providing satisfactory detection results under limited visibility. Finally, the model performs well under nighttime conditions. Despite decreased illumination and the obstruction of road signs, the model can still detect objects on the road, ensuring practicality in nighttime scenarios.

Following a thorough evaluation of the enhanced model's detection capabilities, a decision was made to further assess its performance. For this purpose, additional images were selected, and a comparative experiment was carried out to juxtapose the enhanced model against other models. It's essential to note that all models were subjected to identical parameter settings, featuring a confidence threshold set at 0.25 and an Non-Maximum Suppression (NMS) threshold set at 0.45.

**Figure 11:**
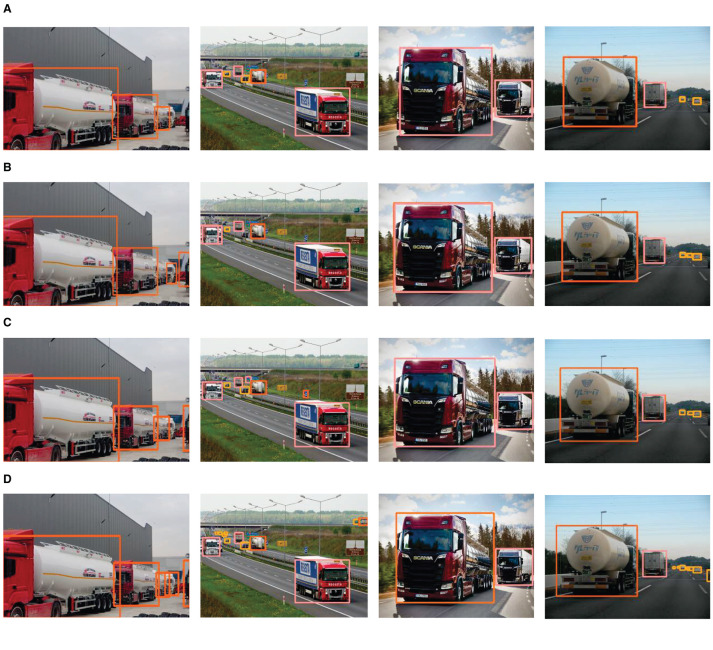
Comparison of detection results. **(A)** Detection results of YOLOv5s. **(B)** Detection results of YOLOv6n. **(C)** Detection results of YOLOv7 = tiny. **(D)** Detection results of HMV-YOLO.

The comparative results, as shown in Figure 11, visually represent the differences in detection performance among the four models.

In the first image, a scenario with densely parked hazardous material vehicles led to a significant loss of distinguishing features. YOLOv5s and YOLOv7-tiny faced challenges in accurately detecting these vehicles, while HMV-YOLO excelled, demonstrating its capability to effectively detect these vehicles and overcome feature loss challenges. YOLOv6n also exhibited some performance in this scene but was slightly inferior to the HMV-YOLO model.

In the second image, a complex highway scene involving multiple object categories was observed. YOLOv5s and YOLOv6n exhibited similar detection performance, both demonstrating limitations in detecting small targets. YOLOv7-tiny encountered issues, misclassifying a signboard and missing vehicle targets on the bridge. In contrast, the HMV-YOLO model excelled, avoiding misclassification and identifying more small target vehicles.

In the third image, the coexistence of a hazardous material vehicle and a truck posed a classification challenge. YOLOv5s, YOLOv6n, and YOLOv7-tiny encountered classification errors. In contrast, the HMV-YOLO model effectively distinguished between these two target categories, showcasing its advanced classification performance.

The last image depicts a highway scene with multiple object categories. YOLOv5s and YOLOv6n accurately classified detected targets but failed to detect more small vehicle targets. YOLOv7-tiny made dual detections on the cargo truck target. In contrast, the HMV-YOLO model accurately classified objects and additionally detected more cars, attributed to the enhanced feature aggregation capability of the LTPAN structure within the HMV-YOLO model.

### 4.4 Ablation experiment

To evaluate the effectiveness of the proposed method, a stepwise integration of the new modules introduced in this paper into the original YOLOv7-tiny model was performed. The impact of each new module on the model's performance was systematically evaluated, and the results are presented in Table 7. In the table, the “√” symbol denotes the incorporation of the respective module from the header row. YOLOv7-tiny uses the LeakyReLU activation function, while the CBSG module utilizes the SiLU activation function. Therefore, experiments were conducted separately with the SiLU activation function. In the third series of experiments, the combination of SiLU and GRN corresponds to the CBSG module. In the fourth set of experiments, SPPFC splices SE modules (Hu et al., 2018) based on SPPF in a residual way to filter useless channel features. The table reveals that the introduction of each proposed method yielded varying degrees of improvement across almost all metrics.

**Table 7:** Ablation experiment results.

**Group**	**SiLU**	**GRN**	**SPPFC**	**LTPAN**	***P***	***R***	**map@.5**	**map@.5.95**
1					0.816	0.789	0.833	0.639
2	√				0.88	0.778	0.848	0.651
3	√	√			0.894	0.775	0.856	0.661
4			√		0.853	0.787	0.846	0.651
5				√	**0.911**	0.744	0.853	0.653
6	√	√	√	√	0.865	**0.801**	**0.863**	**0.663**

Bold values represents the best metrics.

Notably, the introduction of the LTPAN structure led to an impressive Precision metric of 91.1%, marking a substantial 9.5% increase compared to the original model. This underscores the LTPAN structure's superior feature fusion capability when contrasted with the PAN structure.

While the introduction of individual improvement methods may have caused a reduction in the model's Recall metric, it was pleasantly surprising to observe that, when all the methods were combined, the Recall metric increased to 80.1%, surpassing the original model by 1.2%. Although the combined Precision metric did not reach the same remarkable level as when the LTPAN structure was added in isolation, it still outperformed the original model by 4.9%. The improvements in the map@.5 and map@.5.95 metrics were relatively similar, with both exhibiting increases of 3 and 2.4%, respectively, over the original model. These experimental results illustrate the exceptional performance of the proposed methods and their combined application in practical scenarios.

### 4.5 Discussion

The method primarily focuses on integrating new modules to enhance performance, potentially leading to an increase in the model's parameter count. Addressing the challenge of model lightweighting is crucial to ensure the practical deployment of the hazardous material vehicle detection model. Future research should delve into lightweighting techniques, such as quantization and pruning, to reduce the model's size and resource requirements without compromising performance.

Despite achieving effective performance improvements, the enhancement of the model's inference time has not been significantly optimized. Real-time applications require time optimization, and future research should concentrate on model optimization techniques, including hardware acceleration, parallel processing, and model compression, to achieve faster and more efficient hazardous material vehicle detection during the inference process.

Furthermore, although our research has achieved some improvement in accuracy, there is still room for further enhancement. Achieving higher accuracy is crucial in applications where false positives can have serious consequences. Future research should explore advanced training strategies, data augmentation techniques, and fine-tuning methods to further improve the accuracy of the hazardous material vehicle detection model.

Addressing these limitations and conducting future work will not only contribute to refining the hazardous material vehicle detection model but also enhance its practicality and adaptability to widely deployed scenarios.

## 5 Conclusions

This study introduces and systematically evaluates novel modules in the HMV-YOLO hazmat vehicle detection model. The progressive integration of these modules into the original YOLOv7-tiny model results in significant improvements across various performance metrics. Notably, the introduction of the LTPAN structure demonstrates outstanding feature fusion capability, leading to a remarkable 9.5% increase in *Precision* compared to the original model.

While the introduction of individual modules occasionally results in decreases in the model's *Recall* metric, it is encouraging to find that the combined application of all methods results in an overall increase in *Recall* to 80.1%, surpassing the original model by 1.2%. Although the combined *Precision* metric does not reach the same exceptional level as the LTPAN structure in isolation, it still outperforms the original model by 4.9%. Additionally, the *map@*.5 and *map@*.5.95 metrics show consistent improvements, both surpassing the original model by 3 and 2.4%, respectively.

These findings underscore the effectiveness of the proposed methods in enhancing the performance of the hazmat vehicle detection model. The LTPAN structure, in particular, demonstrates its ability to significantly improve feature fusion, making it a valuable addition to the model's architecture. Overall, the combination of these innovations showcases their practical relevance and excellent performance in real-world applications, furthering the field of hazardous material vehicle detection.

## Data availability statement

The original contributions presented in the study are included in the article/supplementary material, further inquiries can be directed to the corresponding authors.

## Author contributions

LW: Conceptualization, Methodology, Validation, Writing—original draft. BL: Conceptualization, Project administration, Validation, Writing—review & editing. WS: Validation, Writing—review & editing. ZL: Data curation, Writing—original draft. KC: Visualization, Writing—original draft. WZ: Supervision, Writing—original draft.
